# Hybrid nanofilms as topical anesthetics for pain-free procedures in dentistry

**DOI:** 10.1038/s41598-020-68247-0

**Published:** 2020-07-09

**Authors:** Lígia N. M. Ribeiro, Michelle Franz-Montan, Ana C. S. Alcântara, Márcia C. Breitkreitz, Simone R. Castro, Viviane A. Guilherme, Bruno V. Muniz, Gustavo H. Rodrigues da Silva, Eneida de Paula

**Affiliations:** 1 Department of Biochemistry and Tissue Biology, Institute of Biology, University of Campinas - UNICAMP, Rua Monteiro Lobato, 255-Cidade Universitária, Campinas, São Paulo 13083862 Brazil; 2 Department of Physiological Sciences, Piracicaba Dental School, UNICAMP, Piracicaba, São Paulo Brazil; 3 Department of Chemistry, Federal University of Maranhão, São Luís, Maranhão Brazil; 4 Department of Analytical Chemistry, Institute of Chemistry, University of Campinas, UNICAMP, Campinas, São Paulo Brazil; 5 School of Veterinary Medicine, Federal University of Uberlândia, UFU, Uberlândia, Minas Gerais Brazil

**Keywords:** Materials science, Nanoscience and technology

## Abstract

Topical anesthetics are widely applied in order to relieve the discomfort and anxiety caused by needle insertion and other painful superficial interventions at the oral cavity. So far, there are no commercially available effective topical anesthetic formulations for that purpose, and the most of developments are related to hydrophilic and low mucoadhesive forms. Therefore, we have prepared different hybrid nanofilms composed of biopolymer matrices (chitosan, pectin, and chitosan-pectin) blended with nanostructured lipid carriers (NLC) loading the eutectic mixture of 5% lidocaine–prilocaine (LDC–PLC), in order to fulfill this gap in the market. These dual systems were processed as hybrid nanofilms by the solvent/casting method, and its mucoadhesive, structural and mechanical properties were detailed. The most appropriate hybrid nanofilm combined the advantages of both pectin (PCT) and NLC components. The resultant material presented sustained LDC–PLC release profile for more than 8 h; permeation across porcine buccal mucosa almost twice higher than control and non-cytotoxicity against 3T3 and HACAT cell lines. Then, the *in* vivo efficacy of PCT/NLC formulation was compared to biopolymer film and commercial drug, exhibiting the longest-lasting anesthetic effect (> 7 h), assessed by *tail flick* test in mice. These pectin-based hybrid nanofilms open perspectives for clinical trials and applications beyond Dentistry.

## Introduction

Topical anesthesia is currently recommended for all dental procedures that can be uncomfortable or painful, principally in the case of needle phobia, pediatric and/or special needs patients^1,2^. Despite this huge demand, there is no available commercial formulation able to eliminate the pain prior to local anesthetics (LA) injection or other superficial procedures in dentistry^3^. EMLA cream is the most successful topical anesthetic for use at the oral mucosa. It is composed of the eutectic mixture of 2.5% lidocaine and 2.5% prilocaine (5% LDC–PLC)^4^. However, this formulation was devised for dermatological administration, and in contact with buccal mucosa causes burning sensation, superficial lesions, bitter taste^3,5^ and inefficient anesthesia in the palatal region^6^. Moreover, in transbuccal anesthesia, there are still some factors, such as saliva lixiviation, swallowing and muscle movements that severely contributed to the failure of formulations processed as gels or creams^3^.

Nanostructured pharmaceutical forms are promising systems designed to overcome the huge lack in the market related to topical anesthetics development^7^. In 2016, we have described detailed structural information regarding the development of nanostructured lipid carriers (NLC) loading LDC–PLC (5%). The optimized formulation exhibited prolonged LDC–PLC release profile and physicochemical stability for 14 months. Conversely, its low viscosity prevented efficient transbuccal application^8^.

In addition to NLC, mucoadhesive biopolymers such as chitosan (CHT) and pectin (PCT) are derived from crabs and apple/citric fruits, respectively. They have been widely used as matrices for drug delivery systems (DDS) due to their abundance, versatility, biocompatibility and biodegradability properties^9–11^. In general, biopolymer films can be easily prepared by the casting-solvent method. They can be produced from different materials and show variable size, thickness and shape, being useful for multiple DDS applications^12–15^. CHT and PCT films improved intestinal/oral mucosa permeability, bioavailability and reduced the cytotoxicity of different classes of drugs^16–20^, It is also known that films and patches are less sensitive to saliva lixiviation than creams and hydrogels when in contact to the oral mucosa^21^. Unfortunately, there are only a few descriptions of polymer films^22–26^ in comparison to hydrogels for topical LA delivery^27^.

The hybridization process is a versatile strategy noticed since primitive civilizations, in which gums, polysaccharides, fats and waxes were combined and used as drugs^28^. Nanostructured hybrid biomaterials combine the main advantages of organic or inorganic nanoparticles (sustained release profile, drug protection, physicochemical stability) with biopolymer matrices (adhesion, suitable mechanical properties, biodegradability) in a single pharmaceutical form^29–31^. Lipid and polymer biomaterials seem to be a brilliant association in the preparation of DDS for unrestricted applications^32^. To date, there are no reports of nanostructured hybrid films developed as topical anesthesia.

In this work, we have prepared mucoadhesive nanostructured films aiming to provide more efficient and less toxic topical anesthetic than the commercially available EMLA cream. Therefore, the systems were composed of hybrid nanofilms containing nanostructured lipid carriers loading 5% LDC–PLC (NLC) incorporated in CHT (2%), PCT (2%) and the polyelectrolyte complex formed by CHT–PCT (2%) solutions. The biopolymer matrices were evaluated in terms of mucoadhesion capacity by the mucin adsorption and mucoadhesive strength tests. The three types of hybrid nanofilms (CHT/NLC, PCT/NLC and CHT–PCT/NLC) were evaluated regarding the in vitro drug release profile and mechanical tests. The most desirable hybrid nanofilm (PCT/NLC) was selected considering its visually appearance, mechanical and physicochemical properties. Then, the structural characterization using FTIR-ATR, DSC and FE-SEM analyses clarified the molecular arrangement of PCT/NLC loading LDC–PLC (5%) hybrid film. This system was also tested for in vitro permeation across porcine oral mucosa. Finally, in vitro safety and in vivo efficacy were confirmed by cell viability (3T3, HACAT) tests and tail flick assay in mice, respectively.

## Materials and methods

### Materials

PLURONIC 188, CHT of medium molecular weight (190–310 kDa) with 75–85% deacetylation degree from crab shells, PCT from citrus fruits, mucin from porcine stomach (type II, bound sialic acid ∼ 1%), Schiff’s reagent 1% basic fuchsine, periodic acid, and sodium metabisulfite were supplied by Sigma (St Louis, MO, USA). LDC and PLC were gifted by Cristália Prod. Quim. Farm. Ltda (Itapira, Brazil). EMLA cream was purchased by AstraZeneca (Brazil). Cetyl palmitate (CP) was donated by Dhaymers Química Fina (Taboao Da Serra, Brazil) and capric/caprylic triglycerides were provided from Lipo do Brasil Ltda (São Bernardo do Campo, Brazil). High-performance liquid chromatography (HPLC)-grade acetonitrile was from J.T. Baker, Phillipsburg, NJ, USA. Deionized water (18 MΩ) was achieved from an Elga USF Maxima Ultra-Pure water purifier (Elga LabWater, High Wycombe, UK).

### NLC preparation method

NLC/LDC–PLC (5%) was prepared through the emulsification–ultrasonication method, as previously described^8^. Briefly, the lipid matrix (cetyl palmitate and capric/caprylic triglycerides) incorporating LDC-PLC (5%) was heated ~ 10 °C above to the solid lipid melting point (~ 64 °C). Simultaneously, the aqueous phase containing PLURONIC 188 was heated to the same temperature and dropped into the oil phase under homogenization by ULTRA-TURRAX, followed by ultrasonication and ice bath up to reach the room temperature. The resulting formulations were sterically stabilized by PLURONIC 188, exhibiting physicochemical stability for 14 months (25 °C).

### Preparation of control and hybrid nanofilms by casting/solvent method

CHT was dispersed in 50 mL of acetic acid 0.1% and PCT was dissolved in 50 mL of deionized water, under magnetic stirring until complete homogenization, up to a final concentration 2% (w/w). Thus, 5% LDC–PLC hydroalcoholic solutions was incorporated in biopolymer suspensions and stirred for 2 h at room temperature. The polyelectrolyte complex between blended CHT (1%) and PCT (1%) were similarly prepared. In the case of hybrid nanofilms, NLC containing 5% LDC–PLC replaced part of the aqueous solutions used for biopolymers, and the samples stirred for 2 h at room temperature. The proportion of NLC/LDC–PLC (loaded with the anesthetics) and biopolymers in the nanofilms was 1:1 (w/w).

Control and hybrid nanofilms were prepared by the casting/solvent evaporation technique^13,33^. The different batches were poured in Petri dishes and dried for 72 h at room temperature. After that, the films were slightly removed and stored.

### Mucoadhesion tests

#### In vitro mucin mucoadhesion test

The mucoadhesive properties of biopolymer matrices (CHT, PCT and CHT-PCT) were quantified by its mucin adsorption capacity^9,34^. A Schiff reagent solution was obtained by the mixture of 30 mL of Schiff reagent with 6 mL of 1 M HCl. Then, 0.1 g of sodium metabisulphite was added to 6 mL of the resultant solution, followed by incubation at 37 °C for 1 h. A solution of periodic acid reagent was prepared with 10 μL of 50% periodic acid solution plus 7 mL of 7% acetic acid.

Standard calibration curves were obtained from mucin standard solutions in the range from 0.25 to 2 mg/mL. The samples were incubated at 37 °C for 2 h in a water bath. Subsequently, 0.2 mL of Schiff reagent was added, and the absorbance of the resultant solutions was measured 30 min later in a UV–vis spectrophotometer (Shimadzu UV1201) at 555 nm. Samples were analyzed in the same procedure: 10 mg of each biopolymer matrix was incorporated in the mucin solutions, stirred and centrifuged at 4,100 g for 5 min. The supernatant was used for the free mucin quantification, determined from the standard curve calibration.

#### Preparation of porcine oral mucosa

Porcine oral mucosa was prepared as previously described^3^. Pig maxillae (from 5-months-old Landrace pigs weighing around 75–80 kg) were acquired in a local slaughterhouse (promptly after slaughter), stored in ice-cold isotonic phosphate buffer at pH 7.4, and conducted to the laboratory within 1 h. For the in vitro permeation test, samples of buccal mucosa from the cheek site (non-keratinized mucosa model) were separated from the underlying tissue through a scalpel and washed with saline solution. Intact mucosa was immersed in deionized water at 60 °C for 2 min. The epithelium was cautiously separated from the connective tissue and immediately used.

In the mucoadhesive strength test, the porcine cheek was removed with a scalpel and washed in distilled water. The underlying muscle layer was smoothed and retained, supporting the mucosa. The tissues were kept in isotonic phosphate buffer at pH 7.4 and were immediately employed as biological barrier in the experiments. The experiments were performed with mucosa from at least three different animals.

#### Mucoadhesion strength test of control and hybrid nanofilm

The mucoadhesion strength of the control and hybrid films were quantified in terms of the force required to detach (DF) them from porcine buccal mucosa, by a texture analyzer (Model TA-XT Plus, Stable Micro Systems), working in texture profile analysis (TPA) mode^35^. The porcine mucosa was horizontally located at the end of TPA probe and the formulation was placed at the upper end. Prior the test, fresh buccal mucosa was hydrated with artificial saliva (50 µL) for 5 min. The probe was then lowered until it made contact with the mucosa surface. The rupture tensile was measured by applying of a compressive force (0.5 N) for 30 s, being moved at a constant speed of 1.0 mm s^−1^. The force necessary to detach the samples from the mucosa surface was determined from the curve of force plotted in contrast to distance. All the analyses were performed at room temperature, n = 5.

#### Mechanical properties of control and hybrid nanofilm

The mechanical properties of control and hybrid films were carried out by a stress–strain test, through a Model 1122 Instron Universal Testing Machine (Instron Corp., Canton, MA, USA), in order to clarify the NLC influence in the biopolymer films. The samples were designed to have rectangular dimensions (100 mm × 5 mm) in agreement to the ASTM D882-97 test^33^. The samples were maintained at 30% RH and 25 °C for 48 h before the analysis. The maximum tensile strength (TS) and maximum percentage elongation at break (EB%) were provided. Films were stretched using a speed of 50 mm min^−1^. Tensile properties were obtained from the plot of stress (tensile force/initial cross-sectional area) *vs*. strain (extension as a fraction of the original length), n = 5.

#### In vitro LDC-PLC release test

The in vitro release of LDC-PLC from the control and hybrid nanofilm was measured through a Franz-cell vertical diffusion system. In the donor compartment, the samples were added as circles with 1 cm of diameter compatible to donor compartment area. The acceptor compartment (4 mL) was filled with 5 mM TWEEN in PBS buffer, pH 7.4, in order to provide sink condition. The compartments were separated by a (10,000 Da molecular exclusion pore size) membrane. 200 µL samples were withdrawn from the acceptor compartment at predetermined intervals and quantified by HPLC (λ = 220 nm). The volume was kept constant by the replacement with buffer solution (200 µL). The two peak areas in the chromatograms were employed to calculate LDC and PLC percentages released, n = 6^8^.

The mathematical modeling of the Kinect curves from the samples was done using KinetDS 3.0 software (Aleksander Mendyk, Kraków, Poland)^36^. Among all the tested models (zero order, first order, Higuchi, Korsmeyer-Peppas and Weibull), Weibull (Eq. 1) was the best fitted model, according to the coefficient of determination (Table S1).

1 $$m = 1 - exp\left[ {\frac{{ - (t)^{b} }}{a}} \right]$$

where *m* is the concentration of LDC-PLC released at the time *t*, *b* is the release exponent, and *a* is the time scale of release.

#### In vitro LDC-PLC permeation across oral mucosa

In vitro LDC-PLC permeation test across porcine buccal mucosa was carried out through a Franz-type vertical diffusion system. The buccal mucosa was located on 0.45 µm cellulose filter, facing the filter with the connective side of the tissue. The samples, buccal mucosa and membrane filter were clamped between the donor and acceptor compartments^3^. The acceptor compartment was filled with filtered and degassed PBS (pH 7.4) plus TWEEN solution (5%, w/v). In order to maintain sink conditions, the concentrations of the drugs in this compartment never reached 10% of the drugs (LDC = 17.67 mg/mL; PLC = 23.14 mg/mL) solubility. The test was performed at 37 °C for 5 h, under magnetic stirring (350 rpm). At determined times, 300 µL of the samples were analyzed by HPLC and the volumes were replaced. The cumulative amounts of the LA permeated across buccal mucosa (per unit of area) were plotted in function of time (n = 6). The steady-state flux of LDC–PLC was estimated from the slopes of the linear portions of the curves. The lag times were calculated from the intercepts on the time axis^37^. The apparent permeability coefficient (P_aap_) was calculated according to the equation bellow^38^:

2 $${\text{P}}_{{{\text{aap}}}} = {\text{ J}}/{\text{AxC}}_{{\text{o}}}$$

where: J is the permeation rate (mg h^−1^), A (cm^2^) is the area of porcine mucosa and C_o_ is the initial concentration of LDC and PLC (mg mL^−1^).

#### Structural characterization of control and hybrid nanofilm

The structural characterization of PCT, NLC loading 5% LDC–PLC, PCT/LDC-PLC and PCT/NLC samples were carried out by FTIR-ATR, DSC, and FE-SEM techniques.

ATR-FTIR spectra were obtained using an infrared spectrometer equipped with ATR (Bruker IFS, Bruker, Billerica, MA, USA), in the range of 4500–500 cm^−1^.

DSC analyses were performed with a TA Q20 calorimeter (TA Instruments, New Castle, DE, USA) equipped with a cooling system. The samples (5 mg) were disposed in aluminum pans and the thermogram were recorded from 0 to 200 °C, at 10 °C/min heating rate, under a flow of nitrogen.

A FE-SEM equipment (FEI-NOVA NanoSEM 230, Sydney, NSW, Australia) was employed to reveal the textural properties of the control and hybrid nanofilm. The samples were attached on a carbon tape and sputtered with gold on the surface.

#### In vitro cell viability test

The cytotoxicity of PCT, 5% PCT/LDC-PLC and PCT/NLC containing 5% LDC-PLC was determined by in vitro cell viability test, using MTT dye and Balb/c 3T3 fibroblasts and immortalized human keratinocytes cells (HACAT).

Concisely, the cells were seeded in 96-well culture plates and incubated for 2 h (37 °C, 5% CO_2_). The Roswell Park Memorial Institute culture medium was changed by 200 µL of fresh medium, with each sample (1 cm^2^). Subsequently to the exposure time (2 h), the medium was removed, and the plate was washed with PBS (pH 7.4). Then, 200 µL of medium containing 0.5 mg/mL of MTT reagent was added to each well, and incubated for 2 h and 24 h at 37 °C. After that, MTT solution was removed and 200 µL of ethanol was added to each well, dissolving the formed formazan crystals. Formazan absorbance was quantified in a microplate reader at 570 nm. Results were provided as the mean viability percentage ± standard error means (SEM), n = 3.

#### In vivo efficacy through tail flick test

The protocol was approved by the UNICAMP Institutional Animal Care and Use Committee (#4468–1/2017), following the instructions of the Guide for the Care and Use of Laboratory Animals. Male adult Swiss mice (25–30 g) were purchased from the *Centro Multidisciplinar para Investigação Biológica na área de Ciência em Animais de Laboratório* (CEMIB-UNICAMP, Campinas, Brazil), and kept (5/cage) with free access to food and water.

For the test, mice were placed in a restraint over an analgesimeter, with a portion of the tail (5 cm from its top) available to heat from a projector lamp (55 °C ± 1 °C). 30 s cut-off times was employed to prevent injury and the baseline (normal response to the noxious stimulus) was measured. For the caudal nerve blockage, samples (1 cm^2^) containing 5% LDC-PLC were administered and occluded on the back of the mice tail. The experiment started 30 min after sample administration and measurements were taken every 30 min during the first 1 h; followed by each 60 min until for 7 h.

### Statistical analyses

The statistical data analyses were carried out using GraphPad Prism v.6.01 (Northampton, MA). Student t-test was employed to evaluate significant differences in the hybrid nanofilms and its respective controls (biopolymers films) in terms of mucoadhesion, mechanical and permeation tests. One-way ANOVA followed by Tukey post hoc multiple comparison tests were used to analyze the data from the in vitro cell viability and tail flick tests. The significance level was defined as 5% (p < 0.05).

## Results

### In vitro mucin mucoadhesion test

**Figure 1:**
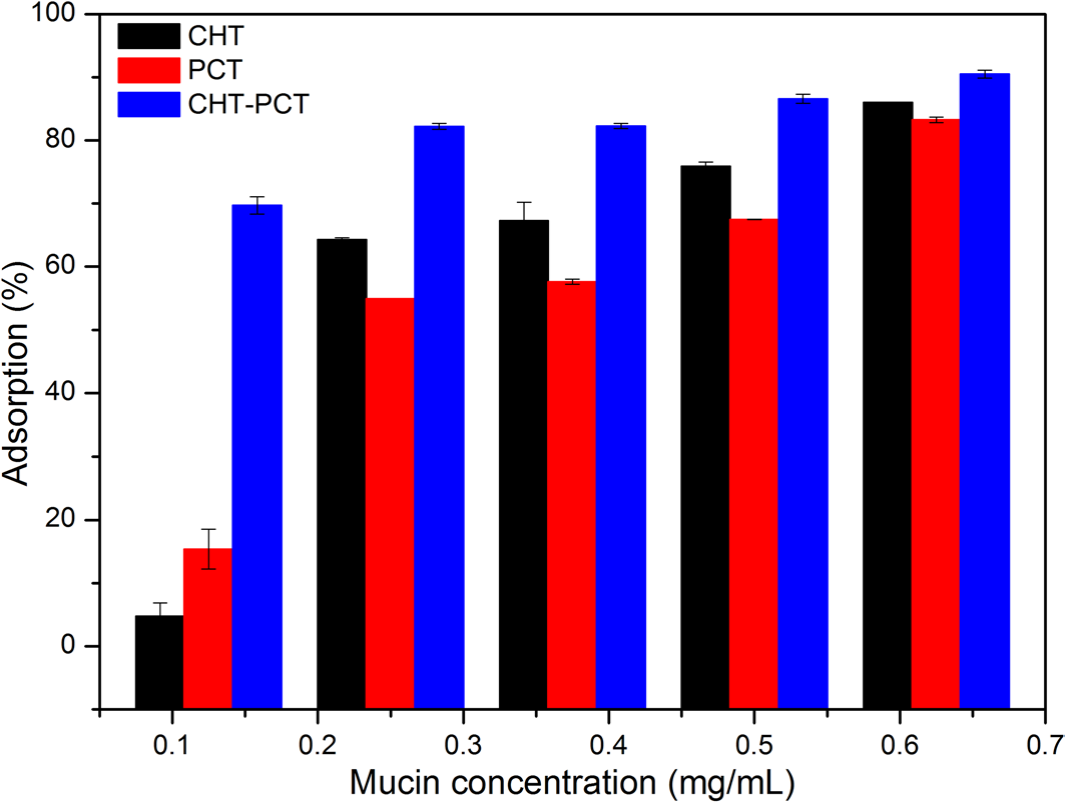
In vitro mucin mucoadhesion test with chitosan (CHT), pectin (PCT) and polyelectrolyte complex (CHT–PCT) matrices. Data are mean ± S.D., n = 3.

Mucoadhesion is a mandatory property for a DDS to be successfully applied to the oral mucosa. In this sense, different biopolymer matrices were firstly screening regarding their in vitro mucin adsorption ability^9^ to contribute as desirable mucoadhesive matrices to the nanofilms. As shown in Fig. 1, all the biopolymers exhibited suitable mucin adsorption results, ranging from 64 to 85% for CHT, 54 to 83% for PCT and around 90% for CHT-PCT matrices.

### Visual inspection of biopolymer and hybrid nanofilms

The biopolymer films (CHT/LDC–PLC, PCT/LDC–PLC and CHT–PCT/LDC–PLC) as controls and hybrid nanofilms (CHT/NLC, PCT/NLC and CHT–PCT/NLC) were successfully prepared through casting/solvent method^13^. All the samples contained 5% LDC–PLC (w/w). In general, CHT-based control films were more rigid than PCT films. The hybrid nanofilms composed of CHT/NLC and CHT–PCT/NLC were friable and the lipid excipient was disposed as white clusters at the biopolymers films surfaces. On the other hand, PCT/NLC hybrid nanofilm showed uniform aspect, suitable flexibility and pectin-based pale orange color (Fig. S1).

### Mucoadhesion strength test of control and hybrid nanofilm

The mucoadhesion strength test is a mechanical analysis that simulates the force (N) required to detach the films from the mucosa surface (detachment force, DF). Table 1 shows that all the hybrid nanofilms exhibited lower DF from oral mucosa than their respective control. PCT/NLC showed highest (p < 0.05) DF values (0.06 N) among all the hybrid nanofilms.

**Table 1:** Mechanical properties of nanofilms in terms of the maximum tensile strength (TS), maximum percentage elongation at break (EB%) and detachment force (DF) from the oral mucosa.

Films	TS (MPa)	EB (%)	DF (N)
CHT/LDC–PLC	2.27 (0.51)	4.58 (0.47)	0.06 (0.008)
CHT/NLC	3.34 (0.92)	6.19 (0.90)*	0.02 (0.006)*
PCT/LDC–PLC	3.16 (1.03)	7.09 (1.31)	0.14 (0.050)
PCT/NLC	2.98 (0.42)	9.45 (0.46)*	0.06 (0.001)*
CHT–PCT/LDC–PLC	1.35 (0.52)	3.75 (1.76)	0.06 (0.030)
CHT–PCT/NLC	1.20 (0.43)	2.13 (0.23)	0.01 (0.001)*

Data are mean ± S.D., n = 5. *t test, p < 0.05. Each parameter was compared with its respective control.

### Mechanical properties of control and hybrid nanofilm

The stress-strained tests in Table 1 provided information regarding the lipid influence in the mechanical properties of biopolymer films. The biopolymer and hybrid films were evaluated in terms of maximum tensile strength (TS) and maximum percentage of elongation at break (EB%). TS values did not significantly change in the nanofilms in comparison to their respective controls (p > 0.05), ranging from 1.20 to 3.34 MPa. On the other hand, EB% values were higher for CHT and PCT hybrid nanofilms than their respective biopolymer films (p < 0.05). PCT/NLC showed the highest EB values (9.45%) among the samples.

### In vitro LDC–PLC release test

**Figure 2:**
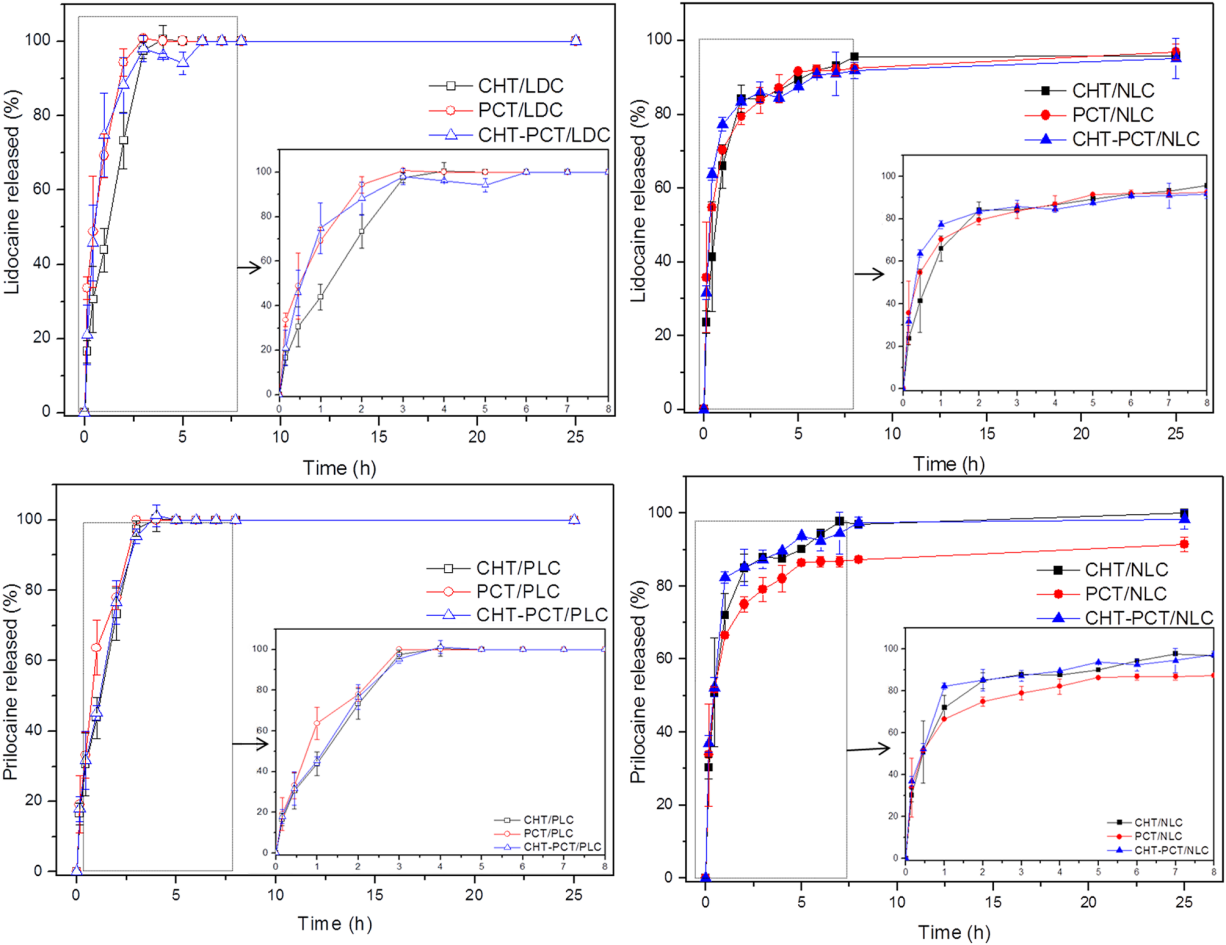
In vitro sustained release profile of lidocaine (top) and prilocaine (bottom) from biopolymers (left) and hybrid nanofilms (right). Data mean ± S.D., n = 6, 37 °C. *LDC*  2.5% lidocaine, *PLC* 2.5% prilocaine, *CHT * 2% chitosan, *PCT* 2% pectin, *CHT–PCT* 2% chitosan-pectin, *NLC* nanostructured lipid carriers encapsulating 5% LDC–PLC.

The LDC and PLC release profile from biopolymers and hybrid films were in vitro evaluated, through vertical Franz diffusion cells and quantified by HPLC for 25 h at 37 °C^2,8^. Figure 2 showed that in the first 5 h of the experiment, all the biopolymer films containing 5% LDC-PLC (controls) released 100% of both LA. Instead, all the hybrid nanofilms were able to prolong the in vitro LDC-PLC release until the end of the experiment. After 8 h of the test, films composed of CHT/NLC released around 96% and 97%, PCT/NLC approximately 92% and 87%, and CHT-PCT/NLC *ca.* 92% and 96%, for LDC and PLC, respectively. These hybrids batches also exhibited burst release effect, releasing both LA in the range of 65–80% in the first 2 h of the experiment. According to Weibull modeling, the release mechanism was a Fickian diffusion, given by the *b* values (0.01 to 0.38)^39^.

Considering the abovementioned results, such as visual appearance, mucoadhesion capacity, mechanical properties and LDC-PLC in vitro release profile, PCT/NLC hybrid nanofilm (Fig. S1) was chosen as the most appropriate sample to be performed in subsequent in vitro and in vivo analyses.

### In vitro LDC–PLC permeation across oral mucosa

Porcine oral mucosa is usually employed as the biological barrier in permeation tests, due to its resemblance with the composition, histology and permeability of human mucosa^37^. The permeation parameters of LDC–PLC from the different films across oral mucosa are displayed in Table 2. LDC and PLC fluxes and apparent permeability coefficient (P_aap_) were almost twice higher in hybrid nanofilm in comparison with biopolymer control film, as already described^40–42^. In general, PLC flux and P_aap_ were slower than LDC in all samples, as previously reported in gels^3^. The lag times noticed for the LDC showed no significant difference between PCT/LDC-PLC and PCT/NLC films (p > 0.05). However, PLC in the hybrid film exhibited an immediate permeation, showing a *lag time* = 0.

**Table 2:** LDC–PLC permeation tests from films through porcine buccal mucosa.

LA	Film	Flux (µg cm^−2^ h^−1^)	P_aap_ (cm h^−1^) × 10^–3^	*Lag time* (h)
Lidocaine	PCT/LDC–PLC	56.53 ± 13.63	2.26 ± 0.55	1.82 ± 1.06
	PCT/NLC	108.76 ± 37.72*	4.35 ± 1.51*	2.05 ± 5.07
Prilocaine	PCT/LDC–PLC	49.96 ± 23.48	1.99 ± 0.94	2.15 ± 1.14
	PCT/NLC	89.73 ± 39.40*	3.76 ± 1.70*	0.00 ± 5.84*

Steady-state flux (µg cm^−2^ h^−1^), apparent permeability coefficient (P_aap_; cm.h^-1^ × 10^–3^) and lag time (h) were quantified by HPLC. Data are mean (± SD), n = 6 (p < 0.05, t test). Each parameter was evaluated separately for each anesthetic.

t test. *Indicate significant statistical difference (p < 0.05) between control and hybrid batches.

### Structural characterization of control and hybrid nanofilm

The structural characterization was carried out through FTIR-ATR, DSC and FE-SEM methods for NLC loading 5% LDC–PLC, hybrid and control PCT films, in order to obtain information regarding the molecular arrangement of PCT/NLC and possible interactions among the excipients.

**Figure 3:**
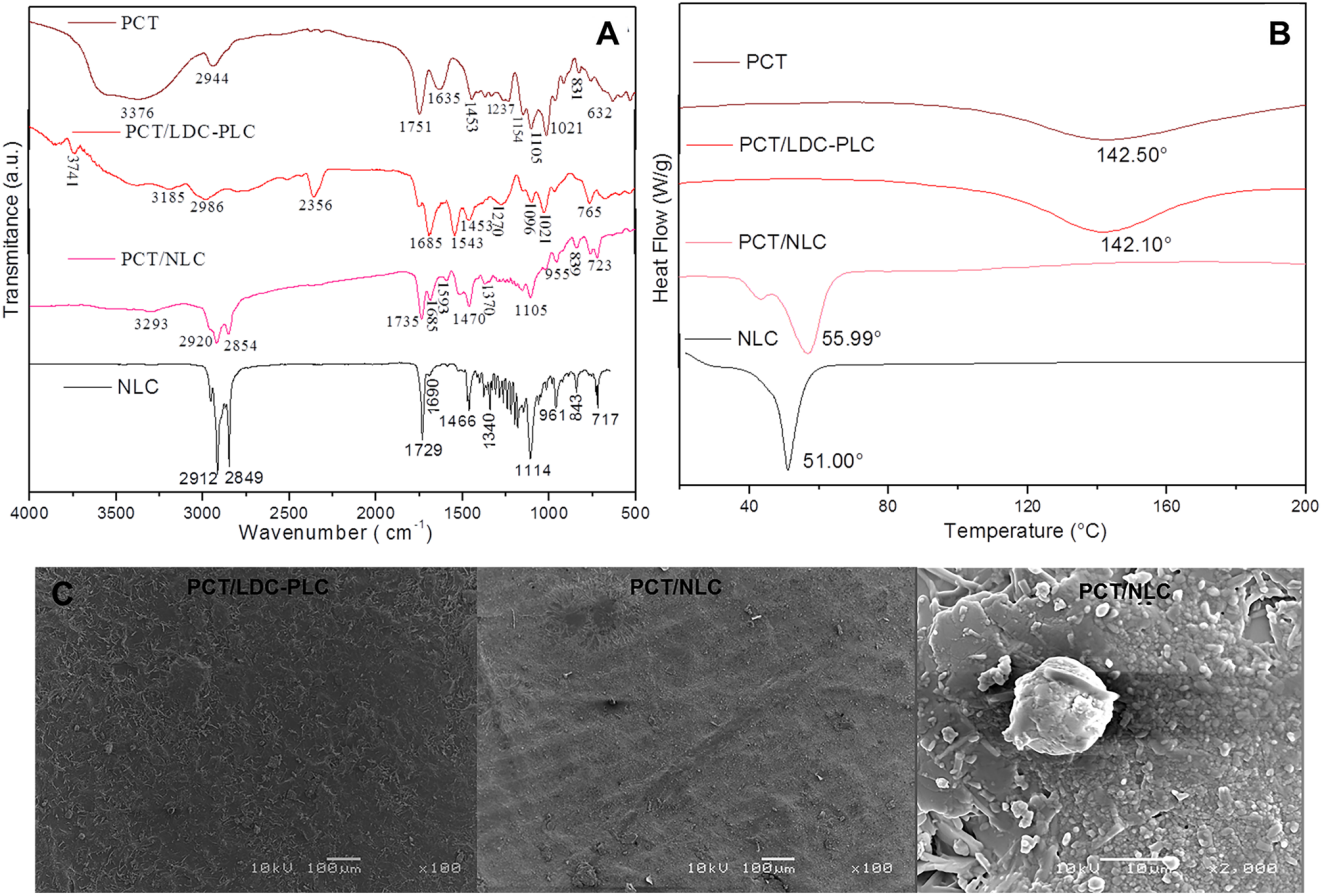
Structural characterization of pectin (PCT), control film (PCT/LDC–PLC) and hybrid (PCT/NLC) film and NLC loading 5% LDC–PLC, in terms of FTIR-ATR (**A**), DSC (**B**) and FE-SEM (**C**) techniques. The employed magnifications are displayed in the images.

FTIR-ATR analysis (Fig. 3A) shows the characteristic bands of NLC encapsulating 5% LDC–PLC, PCT, hybrid and control films containing the anesthetics. The spectroscopic profile of the excipient and drugs that composed NLC were already described^8^. In this case, PCT and PCT/LDC-PLC spectra revealed polysaccharide bands in the region of 3185–3776 cm^−1^ and 1635–1685 cm^−1^, ascribed to the ν_OH_ and asymmetric stretching vibration of –COO^−^ groups, respectively^9^. In the PCT/NLC and NLC spectra, were observed a set of bands related to NLC spectroscopic profile, such as the bands at 2920 and 2912 cm^−1^, 2854 and 2849 cm^−1^_,_ 1735 and 1729 cm^−1^, and 1105 and 1114 cm^−1^ corresponding to the ν_CH_, ν_O–CH2_, ν_C=O_, and ν_C=O_ vibration mode^8^. Moreover, it was observed that the bands ascribed to carboxylate and hydroxyl vibration modes of PCT at 1635 and 3376 cm^−1^ are shifted toward lower wavenumber values in PCT/NLC spectrum (1593 and 3293 cm^−1^, respectively), due to the existence of hydrogen bonding interaction involving these groups between the organic moieties.

In DSC analyses (Fig. 3B), samples were subjected to a controlled heating flow, and their thermodynamic properties were measured. The thermodynamic profiles of LDC, PLC and NLC-based excipients have been previously provided^8^. PCT and PCT/LDC-PLC calorimetry showed endothermic peaks centered at 142.50 °C and 142.10 °C, respectively, related to the biopolymer melting point^9^. In contrast, in the hybrid nanofilm and NLC curves were detected endothermic peaks centered at 55.99 °C and 51.00 °C, related to NLC excipients melting point^8^.

In the FE-SEM images (Fig. 3C), the surfaces of control and hybrid nanofilms were clearly differentiated. PCT/LDC–PLC samples showed LA crystals superficially arranged in the film surface, while PCT/NLC samples exhibited a rougher surface, in which it was not possible to differentiate the biopolymer and lipid counterparts of the hybrid nanofilm. In higher magnification (2,000 ×), it was also evident the existence of spherical structures (1–10 μm) related to PCT-coated lipid nanoparticles in the hybrid film surface.

### In vitro cell viability test

**Figure 4:**
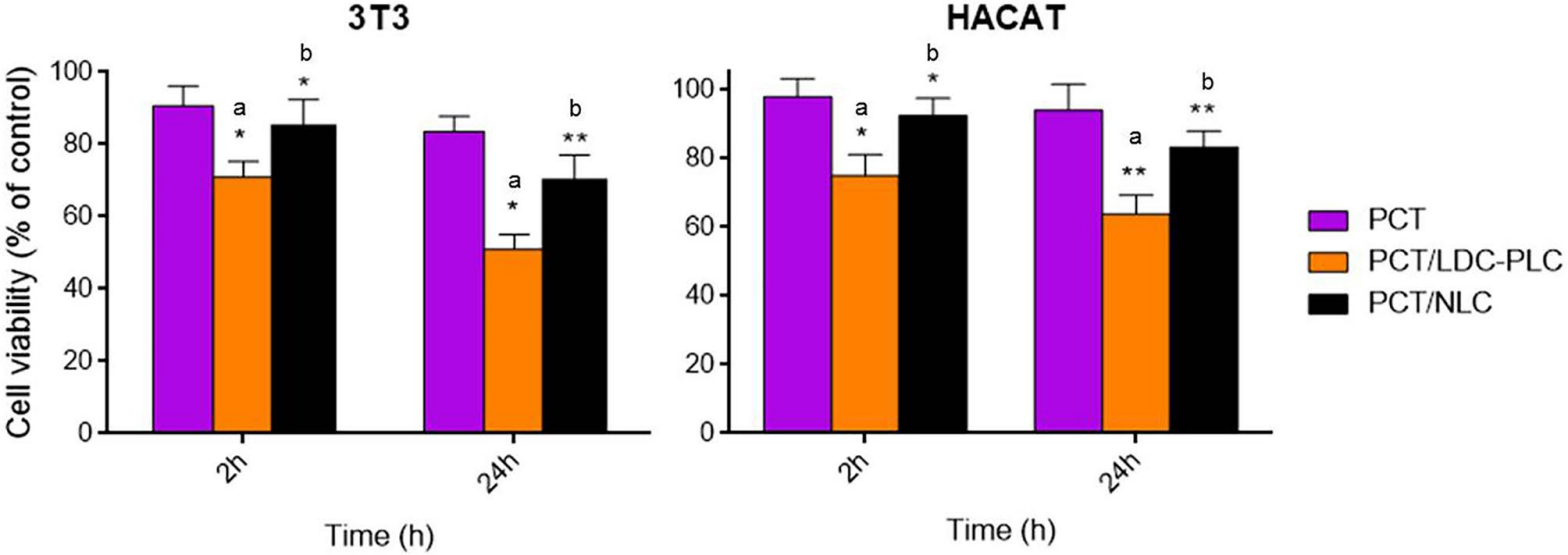
In vitro cell viability (MTT) test in 3T3 (left) and HACAT (right) cells pretreated with pectin (PCT), control (PCT/LDC–PLC) and hybrid (PCT/NLC) films (1 cm^2^). Data are mean ± S.D. (n = 3). Statistical analyses were carried out by one-way ANOVA/Tukey post hoc tests, *p < 0.05, **p < 0.01. a, PCT/LDC-PLC × PCT; b, PCT/LDC-PLC × PCT/NLC.

The potential cytotoxicity of the control and hybrid films was assessed trough in vitro cell viability tests (Fig. 4) in 3T3 and HACAT cell lines. The samples (1cm^2^) were analyzed after 2 h and 24 h of treatments. Pure PCT was used as a control, due to its well-known biocompatibility^43^.

PCT/NLC did not show statistically significant difference from PCT in any of the cell lines and conditions (2 h or 24 h treatment). At the end of the experiment, the viable cells were higher than 70% in both cell lines tested, confirming the biocompatibility of the system. On the other hand, PCT/LDC–PLC caused a significant decrease (p < 0.05) of viable cells in all tests.

### In vivo anesthetic effect through tail flick test

**Figure 5:**
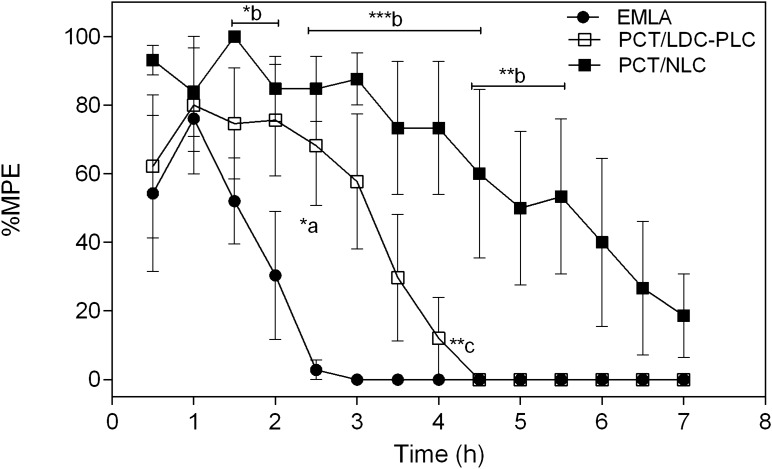
Anesthetic effect (tail-flick) test in mice treated with PCT/LDC-PLC, PCT/NLC and EMLA cream, containing 5% LDC-PLC. Results were expressed as maximum possible effect (%MPE) *vs* time (h). Data are mean ± SEM (n = 6). Statistical analyses were carried out by one-way ANOVA plus Tukey post hoc: a, PCT/LDC-PLC × EMLA cream; b, PCT/NLC × EMLA; c, PCT/LDC–PLC x PCT/NLC (*p < 0.05, **p < 0.01, ***p < 0.001). *PCT* pectin, *LDC–PLC*  5% lidocaine-prilocaine, *NLC* nanostructured lipid carrier loading 5% LDC–PLC.

The tail flick test was performed to measure the antinociception capacity of the hybrid nanofilm in comparison to control (PCT/LDC–PLC) and EMLA cream, in mice. The results in terms of maximum possible effect (%MPE) in Fig. 5 disclosed the longest anesthetic effect of PCT/NLC in comparison to both controls. After 4 h, PCT/NLC exhibited *ca*. 73% MPE, against 12% of PCT/LDC-PLC and no anesthetic activity of EMLA. At the end of the experiment (7 h), only the hybrid nanofilm still displayed some antinociceptive effect (~ 19%).

## Discussion

Mucoadhesion is a multifactorial and complex property, essential for the success of orally administered pharmaceutical forms^44^. Since biomaterials interact with the mucosal tissues through different mechanisms, it is essential to analyze DDS mucoadhesive properties trough different methods. Here, we have employed both chemical (mucin adsorption) and mechanical (mucoadhesion strength) techniques to understand the potential mucoadhesion ability of the prepared nanofilms.

CHT and PCT-based matrices exhibited relevant in vitro mucoadhesion, as seen by the high levels of mucin adsorption, which is the most abundant glycoprotein of the oral mucosa^45^. CHT is the gold standard mucoadhesive biopolymer, providing electrostatic interaction between its positively charged amine groups and the available carboxyl groups of mucin^46^. On the other hand, CHT is soluble in acidic medium and practically insoluble at pH > 6.5, has undesirable taste and can cause allergy^47^. Moreover, its incompatibility with lipid nanoparticles has been already described^2,48^. In here, NLC increased the intrinsic hydrophobicity and hardness of CHT-based films, resulting in heterogeneous and friable forms that contraindicate its use as DDS for the oral mucosa. However, other applications, e.g. for intestinal mucosa treatment, can be further explored.

PCT is another mucoadhesive biopolymer misemployed at the oral mucosa, even though it has satisfactory aqueous solubility and pleasant flavor^49^. The interaction between PCT and mucin is driven by the formation of hydrogen bonds between their free carboxylic acid groups^50^. In addition, the pH of the oral cavity (~ 7.4) and saliva contribute to PCT network relaxation and rapid swelling of the films^51^, fixing it at the site of interest. This was confirmed by its high DF values from oral mucosa (PCT kept attached for 24 h, *data not shown*). In fact, mechanical properties data evidenced that PCT-based films were more flexible than CHT films. This flexibility is an important requisite to improve the patient compliance, once the oral mucosa is a very sensitive tissue.

The hybridization process and film preparation method did not disrupt the lipid nanoparticle in the hybrid film, corroborating the sustained in vitro release profile observed for both LA in the PCT/NLC. Besides, NLC incorporation did not affect the tensile strength of pristine PCT, improving its resistance to rupture. In addition, the distinct PCT/NLC surface in comparison to control showed by FE-SEM (Fig. 3C) indicated that a novel system was formed, in agreement with the structural characterization analyses. As expected, the thermodynamic transitions (DSC) and major FTIR-ATR vibrations modes of NLC were detected in the hybrid nanofilm^8^, since the lipids are the major component of the nanoparticles. As for PCT, its main structural contribution was recorded by the available carboxylate and hydroxyl groups detected by FTIR-ATR in PCT/NLC spectrum (at 1593 and 3293 cm^−1^, respectively), which conferred the mucoadhesion property to the films.

The NLC component in PCT-based film was also responsible for doubling the anesthetics permeation indexes (fluxes and apparent permeability coefficients) in comparison to the control film, with an immediate onset for prilocaine, as required in dentistry clinical practice. Such increased permeation parameters values can explain the prolonged in vivo anesthetic effect of PCT/NLC. This occurred due to the huge contact surface area of NLC, facilitating the passage of nanoparticles across the epithelial barrier^40,42^. NLC were also involved by PCT film, and therefore, LA had a double physical barrier^2^ to overcome, contributing to their non-cytotoxicity, in both cell lines tested. In fact, in a previous work of ours, the cytotoxicity of EMLA cream (containing 5% LDC–PLC) was tested in HaCat cell, showing an IC_50_ after 3 h of treatment^3^. In here, the IC_50_ of PCT/LDC–PLC was noticed only after 24 h of treatment. The cytoprotective effect of biopolymer matrices was expected^2^.

This novel technology can directly impact the industry interests and the dental health quality of patients. In general, many dentists do not purchase commercial topical anesthetics due to their inefficiency; or have to invest in expensive tools, such as nitrous oxide pre-anesthesia, in order to provide less painful oral procedures to the patients. In addition, patients commonly search for dental treatment in critical conditions, due to phobia and fear of oral mucosal injections, what increases the need of invasive interventions and dental extractions. Therefore, these hybrid nanofilms that have proven to be effective, easy handling and cheaper than commercial forms, will supply the lack of marketed pre-anesthesia. This will improve the patient compliance to the treatment, preventing early tooth lost. It should be also noticed that processing of hybrid nanofilms is non-toxic and waste-free, other current concerns for both industry and the consumers.

The abovementioned achievements, especially regarding the safety and in vivo long-lasting anesthetic effect, increase the possible PCT/NLC applications range. The hybrid film can be applied for pain management of oral superficial lesions and incisions, simple extraction of primary teeth, as well as for postoperative pain control^52^. PCT/NLC films can be useful in other mucosal tissues, in dermatology or cosmetology. This hybrid system was patented as a nanotechnological pharmaceutical product in 2017^53^. It will be submitted to clinical trials and investigated for needle-free anesthesia, the Holy Grail of Dentistry Anesthesiology.

## Conclusions

The dentistry procedures can be painless in a near future, which means that the dental treatments will not be delayed, can often prevent unnecessary tooth extraction and surgeries. However, the lack of any effective commercially available pre-anesthetic for dentistry purposes has encouraged this research. We have described the preparation of nanohybrid films for the delivery of lidocaine-prilocaine (5%, w/w). The most effective formulation was based on pectin (2%) as biopolymer and nanostructured lipid carriers encapsulating 5% LDC-PLC in the lipid matrices. The set of structural characterization provided information regarding the molecular arrangement of the novel pharmaceutical form that combined the advantages of excipients, resulting in a flexible, resistant and mucoadhesive hybrid nanofilm. The in vitro LDC-PLC release profile was prolonged for approximately 8 h. This was followed by higher drug permeation values across porcine oral mucosa and longest-lasting anesthesia: more than 7 h in mice, 3 times longer than control film (PCT/LDC–PLC) and the commercial cream, without compromising its safety. This advanced nanostructured material should be now clinically tested, as well as its applications in other fields than dentistry.

## Supplementary information

Supplementary file1 (DOCX 1550 kb)

## Data Availability

All data generated in this work are included in the manuscript.
