# Supplementary material for: Hybrid nanofilms as topical anesthetics for pain-free procedures in dentistry
Source: Sci Rep. 2020 Jul 9;10:11341. doi: 10.1038/s41598-020-68247-0 (PMC7347607; doi:10.1038/s41598-020-68247-0)
Supplement: Supplementary file 1 — Supplementary file1 (DOCX 1550 kb) [file 41598_2020_68247_MOESM1_ESM.docx]

**Supplementary Material**

**Title: Hybrid Nanofilms as Topical Anesthetics for Pain-Free Procedures in Dentistry**

Lígia N. M. Ribeiro^a,e^*, Michelle Franz-Montan^b^, Ana C. S. Alcântara^c^, Márcia C.

Breitkreitz^d^, Simone R. Castro^a^, Viviane A. Guilherme^a^, Bruno V. Muniz^b^, Gustavo H.

R. da Silva^a^ and Eneida de Paula^a^


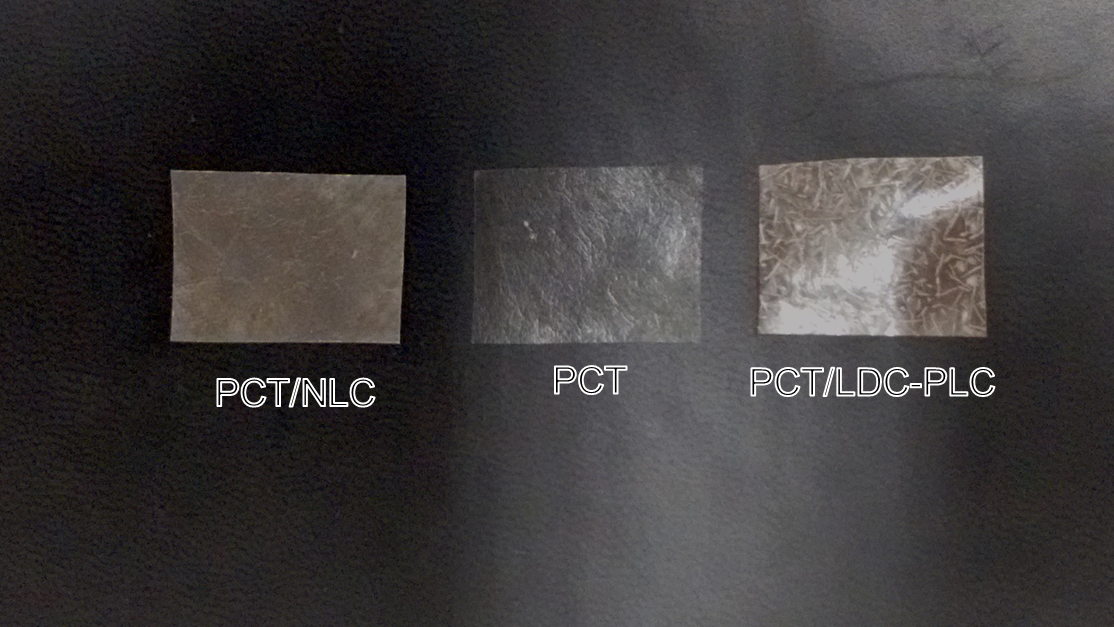


**Figure S1-** Digital photos of PCT/NLC (LDC-PLC, 5%), PCT and PCT/LDC-PLC freshly prepared nanofilms.

**Table S1.** Mathematical modeling of Kinetic curves from the hybrid nanofilms according to the best fitted Weibull model.

| Film/LA | * Weibull | |  |  |
| --- | --- | --- | --- | --- |
|  | R^2^  b | |  |  |
| CHT/NLC-LDC | 0.92 0.06 |  | |  |
| CHT/NLC-PLC | 0.87 0.08 |  | |  |
| PCT/NLC-LDC | 0.83 0.20 |  | |  |
| PCT/NLC-PLC | 0.95 0.03 |  | |  |
| CHT-PCT/NLC-LDC | 0.97 0.38 |  | |  |
| CHT-PCT/NLC-PLC | 0.94 0.22 |  | |  |

R^2^ = determination coefficient; b= curve shape parameter.
